# Development of a process model of posttraumatic growth in psychosis: a qualitative study

**DOI:** 10.3389/fpsyt.2026.1774487

**Published:** 2026-03-30

**Authors:** Fiona Ng, Benjamin-Rose Ingall, Susie Booth, Matt Geeleher, Julian Harrison, Mark Holden, Felix Lewandowski, Yael Mazor, David Roe, Mike Slade

**Affiliations:** 1 School of Health Sciences, Institute of Mental Health, University of Nottingham, Nottingham, United Kingdom; 2 Evaluating Positive Changes in Psychosis (EPOCH) Lived Experience Advisory Panel, Nottingham, United Kingdom; 3 Ono Academic College, Kiryat Ono, Israel; 4 Department of Community Mental Health, University of Haifa, Haifa, Israel; 5 Department of Psychiatric Rehabilitation and Counseling Professions, School of Health Professions, Rutgers University, Newark, NJ, United States; 6 Nord University, Faculty of Nursing and Health Sciences, Health and Community Participation Division, Namsos, Norway

**Keywords:** mechanisms, mental health, outcomes, positive change, posttraumatic growth, psychosis, qualitative, recovery

## Abstract

**Introduction:**

Some people with psychosis experience posttraumatic growth (PTG). PTG is defined as positive psychological changes which occurs after the experience of trauma or adversity, following an emotive struggle with the experience. There is some research into the process and domains of PTG in psychosis. However, limitations of the existing evidence base include a focus on experiences of first-episode psychosis, exclusive inclusion of participants who currently use clinical mental health services, and an emphasis on individual-level facilitators. Increasingly, psychosis is considered as a dimensional construct. The aim of this study was to address these gaps by using a dimensional understanding of psychosis to investigate the process of PTG in psychosis, and to validate the PROSPER framework.

**Method:**

Semi-structured qualitative interviews about the experience of PTG were conducted with 25 individuals with diagnosed or self-reported experiences of psychosis who self-identify with having experiences of PTG. Inductive and deductive thematic analysis was used to identify PTG processes, mechanisms and outcomes.

**Results:**

Participants described the experience of psychosis as a disruption to an individual’s life course, which is moderated by personal and trauma factors. All participants could describe experiences of PTG, but some participants reported difficulty identifying current experiences of PTG, indicating that PTG is a dynamic process. The process from the experience of psychosis to PTG was influenced by two mechanisms: cognitive factors and social/societal factors. All seven domains of the PROSPER framework were validated.

**Conclusions:**

Experiencing PTG in psychosis is possible. Further research to quantify the effects of mechanisms utilising longitudinal designs would assist to strengthen the evidence base. Interventions to support PTG in psychosis are indicated, targeting both individual and population levels.

## 1 Introduction

Personal mental health recovery refers to an individual’s personal definition which may include the ongoing experience of difficulties associated with symptoms (1). A focus on personal definitions of recovery is important as these often contrast to the goals set within medical model frameworks (e.g. symptom amelioration, return to prior functioning). Domains of personal recovery commonly reported by people with mental health problems include, connectedness, hope, identity, meaning, and empowerment (2). Internationally, personal recovery has been recognised within policy and clinical practice (3).

Posttraumatic growth (PTG) has been implicated as part of the mental health recovery process (4). PTG describes an individual’s experience of positive psychological changes following significant trauma (5). The experience of PTG is dialectical. Empirically, studies have demonstrated the inverse relationship between posttraumatic stress and PTG, such that stress can lead to either posttraumatic stress or PTG. Theoretically, the integration of new trauma information involves two processes; assimilation and accommodation (6). Assimilation refers to the integration of new trauma information into existing worldviews (7). This occurs at the time of the trauma and is described as a coping process. Contrary, accommodation involves a changed worldview to incorporate the new trauma information, often involving the creation of new meaning (7). Whilst individuals are likely to experience a combination of both processes, accommodation must occur for people to experience PTG (6). In broader trauma experiences, five domains of PTG are commonly reported: appreciation of life, relationships with others, new possibilities in life, personal strength, and spiritual change (8).

Mixed-method studies on the experience of PTG in psychosis have been conducted. A one year Singaporean longitudinal study of 99 clients with first episode psychosis identified that personal recovery was associated with growth (9). A longitudinal qualitative Canadian study of people accessing first episode psychosis services found that individuals reported both adversity (e.g. trauma, oppression) and growth (e.g. improved sense of self, relationships) through resources (personal, social, community based). This was further confirmed through a one-year US-based qualitative longitudinal study of 43 individuals which found that people not only cope with mental health-related distress but also develop and grow (10). Having hope and resilience was critical to personal growth (10), which has been theoretically (2, 11) and empirically (12, 13) supported by the wider literature. A seven language systematic review of 37 papers found seven domains of PTG; Personal strength and identity, Receiving support, Opportunities and possibilities, Strategies for coping, Perspective shift, Emotional experiences, and Relationships, giving the acronym PROSPER (14). Cross-sectionally, moderate to high PTG has been reported by 50-75% of individuals with psychosis using mental health services (15), with having meaning in life, effective coping skills (16, 17), and clarification of core beliefs (18) mediating the relationship between psychosis symptoms and PTG. The relationship between PTG and actual self-disclosure was mediated by an individual’s level of personal recovery.

There are three limitations with the current PTG in psychosis literature. First, research has predominately focused on first episode psychosis and participants recruited through clinical services. Whilst this provides greater clinical validity, there is increasing evidence that psychosis is a dimensional experience where individuals self-report psychosis symptoms or have differing conceptualisations of their experiences (19). Second, influences of PTG has been studied at individual level factors (e.g. symptoms, trauma type, demographics) to predict growth, placing emphasis on an individual’s ability to grow and overcome adversity using personal resources. Increasingly it is acknowledged that ecological contributions (e.g. relationships, system interactions, policy, cultural/societal scripts) contribute to mental health and personal growth (20). Third, the PTG literature conceptualises trauma as a linear, single, and discrete event. Yet, trauma in psychosis is dynamic and cumulative, occurring across the life course (e.g. childhood trauma), through iatrogenic harm from health service utilisation, and intersectionality experiences. It is unknown whether current knowledge surrounding PTG in psychosis is generalisable to a broader psychosis experiences. Therefore, ecological understanding of how PTG in psychosis occurs and validation of the PROSPER model in a diverse group of people with psychosis.

To address these knowledge gaps, we conducted a qualitative study to explore the experience of PTG in psychosis. There were three objectives to this study:

Objective 1: To investigate the process by which an individual experiences of PTG following psychosis,
Objective 2: To identify the mechanisms which support posttraumatic growth in psychosis, and
Objective 3: To validate the PROSPER framework.

## 2 Methods

This interview study was conducted as part of the Evaluating Positive Changes in Psychosis (EPOCH) Programme, which is investigating how PTG occurs in people with experience of psychosis to develop a digital intervention. Ethics approval was obtained from the University of Nottingham Faculty of Medicine and Health Sciences human ethics committee (FMHS 37-0722). All participants were provided a study information sheet and audio-recorded verbal consent was received prior to the start of the interview.

### 2.1 Participants

Eligibility criteria included: formal diagnosis or self-reported experience of psychosis; experience of positive change or PTG attributed to psychosis experiences; aged over 18 years; resident in England; able to participate in an English-language interview or via a translator; able to provide online informed consent. Self-reported experiences of psychosis include, self-report of previous experiences of psychosis, individuals reporting a clinical diagnosis of a psychosis spectrum disorder, or alternative conceptualisations of psychosis including hearing voices, non-ordinary states, and spiritual emergence.

Recruitment occurred through five routes: social media (X, Facebook), mental health and non-government organisations with established relationships with the research team, study participant recruitment websites (Call for Participants, MQ Mental Health), snowball sampling, and targeted sampling of non-cisgender individuals.

### 2.2 Procedures

All recruitment materials directed potential participants to the participant information sheet hosted on Microsoft Forms. Recruitment material referred to the study as looking for participants who have experienced positive change due to psychosis experiences. Then potential participants completed the eligibility checking questions and provided an email address for contact. Informed consent was obtained prior to the start of the interview. Interviews were conducted via Microsoft Teams or telephone.

Semi-structured interviews were conducted by two researchers (BRI/FN). The interview topic guide and distress protocol were developed in collaboration with five members of the EPOCH Lived Experience Advisory Panel (LEAP) who have experience of psychosis and/or PTG. The topic guide consisted of open questions regarding psychosis experiences, PTG, and relevant prompts. Given the definition of PTG involves the positive psychological changes, the term ‘positive change’ was used interchangeably with ‘PTG’. Participants were reimbursed £20. Interviewers wrote field notes, including the interviewers initial impressions as part of their reflexive practice. The topic guide is shown as Appendix 1.

### 2.3 Analysis

An inductive interpretative approach to thematic analysis was used to explore the process (Objective 1) and the mechanisms (Objective 2) of PTG that was related to the experience of psychosis. To establish the validity of the PROSPER framework (Objective 3), an inductive and deductive approach to thematic analysis was used, with the PROSPER framework acting as the preliminary deductive framework which was refined throughout the analysis process.

Analysis was guided by a six-step process (21). First, interviews were transcribed verbatim and pseudonymised. Second, analysts (BRI, FN) familiarised themselves with three data sources; transcripts for inductive analysis, field notes, and the PROSPER model (14) (used as the deductive framework). Third, three transcripts were coded to develop the inductive coding framework for Objectives 1 and 2, and refined the deductive coding framework for Objective 3. Fourth, discussions were held to compare coding. Disagreements were resolved via consensus, and the coding framework was refined. Fifth, the refined coding framework was discussed with the wider research team (*n* = 5) and the EPOCH LEAP. Further refinements to the coding framework were made by FN. Discussions focused on the refinement of coding and definitions, where disagreements were resolved via consensus. Finally, the refined inductive and deductive coding frameworks were used in coding of the remaining transcripts allowing for further refinements when required. A reflexive approach to the study was used, where researchers directly involved in the data collection and analysis brought different professional and lived experience expertise to the study. One researcher had professional expertise in mental health services research, mental health recovery and PTG, whilst the other brought experience in mental health recovery, and as a person with neurodiversity and LGBTQ+ identity. Both researchers were from a non-clinical background without direct personal experience of psychosis. To ensure that interpretations were grounded in lived experience, findings were discussed with the EPOCH LEAP who supported the interpretation of findings. All coding was conducted using NVivo 12.

The presence of PTG was guided by three elements. First, the values underpinned by the mental health recovery paradigm. Given that the mental health recovery values individual’s as experts of their own experiences, we therefore considered an individual to have experience PTG when they reported this. Second, the definition of PTG refers to the experience as a positive psychological change which implies that PTG is an internal subjective experience. Third, systematic review evidence that has identified seven domains (PROSPER framework) associated with PTG in psychosis (14). Therefore, combining these three elements together, we considered individuals as having experienced PTG when 1) participants self-reported PTG and 2) the change was internally-oriented, or 3) participants endorsed domains as part of the PROSPER framework.

## 3 Findings

Fifty-eight individuals expressed interest in the study, and 28 consented to be interviewed. Three transcripts were excluded due to withdrawal of consent, poor audio quality, or lack of engagement. The analysis included 25 transcripts. Demographic and clinical characteristics of participants are presented in **Table 1**.

**Table 1:** Demographic and clinical characteristics (*N* = 25).

Characteristic	n	%
Age		
18-30 31-45 46-60 61+	8 9 3 5	32 36 12 20
Gender		
Female Male Non-Binary	16 7 2	64 28 8
Relationship Status		
In a relationship Single Prefer not to say	14 9 2	56 36 8
Ethnicity		
Asian/Asian British Black/African/Caribbean/Black British Mixed/Multiple Ethnic Groups White British/White Prefer not to say	4 5 2 13 1	16 20 8 52 4
Highest Qualification		
O levels/GCSE A levels/AS levels/NVQ or Equivalent Degree level Higher degree level	2 8 7 8	8 32 28 32
Employment Situation		
Employed (Full time, Part time, Self) Retired Training and Education Unemployed Other	17 2 1 4 1	68 8 4 16 4
Formal Diagnosis of Psychosis Spectrum Disorder		
Yes No Prefer Not to Say	18 6 1	72 24 4
Current Mental Health Service Use		
Yes No	10 15	40 60

### 3.1 Objective 1: process of PTG in psychosis

**Figure 1:**
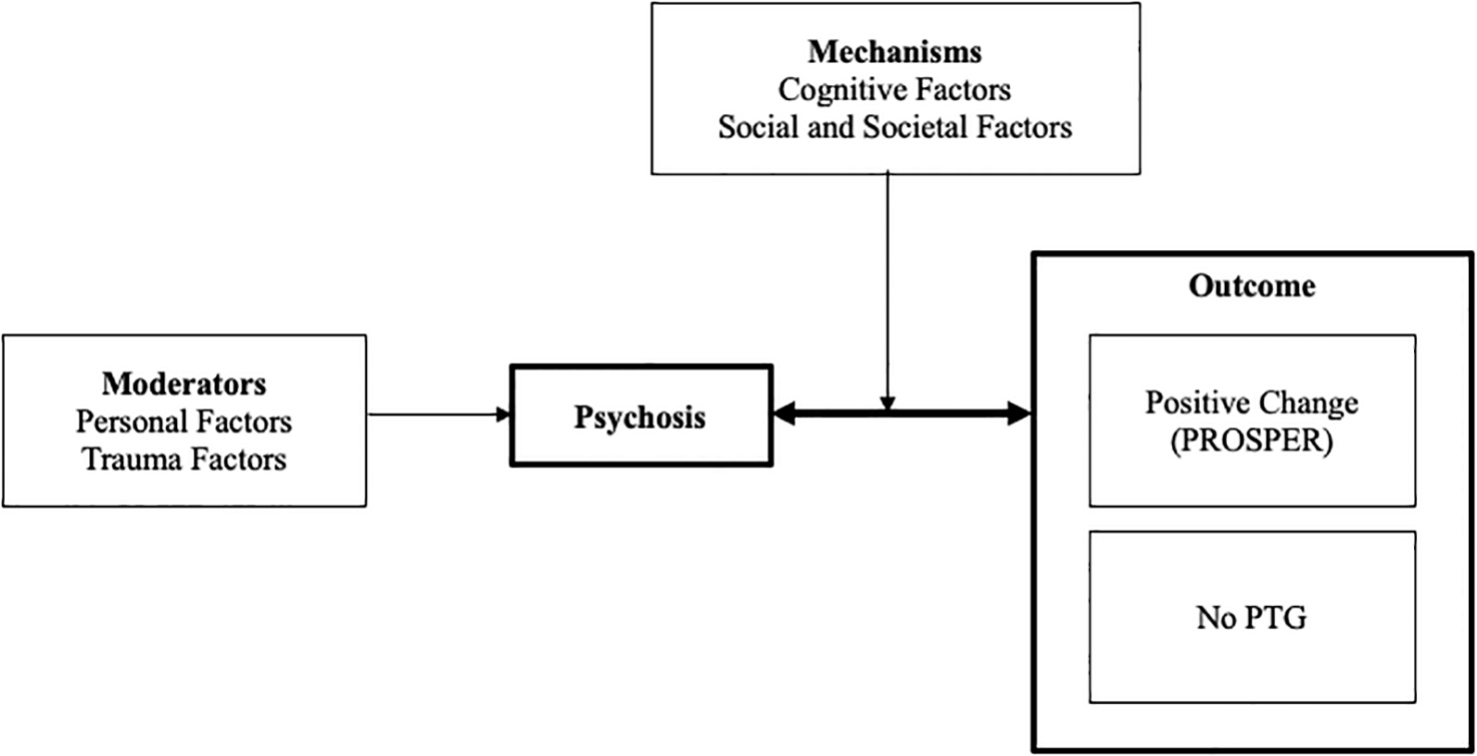
EPOCH process model of PTG in psychosis.

The EPOCH Process Model (**Figure 1**) describes psychosis as a disruption to an individual’s life course and mental turmoil. Two factors moderated psychosis experiences, whilst two factors mediated PTG experiences. PTG was described as dynamic and dependent upon the participant’s circumstances and emotional experiences. Indicators of PTG in psychosis were described through seven factors or not currently experiencing PTG. The full codebook with illustrative quotes is presented as Appendix 2.

All participants viewed psychosis as a disruption to their expected or desired life course, whilst some indicating significant mental turmoil as a result (n=21). Experience of an altered life course was attributed to negative experiences of symptoms, difficulties with processing experiences, making meaning, hospitalisation, missed career paths and isolation. Negative perceived attitudes from self and others exacerbated these factors.

‘I come from a family where it’s very stigmatised … you hear a lot of really strong words around people’s perception on mental health’ (Olivia)

Disclosure affected the experience of direct and self-stigma (n=6), where the response to disclosure was influenced by societal and individual understanding of psychosis. Examples of direct stigma included relationship loss, bullying, and being perceived as different or dangerous. Self-stigma experiences included fear of being victimised or judged.

‘I couldn’t tell someone my life story because people wouldn’t understand. I’d have to really build up that trust with them…’ (Bella)

Personal and trauma factors moderated the experience of psychosis (see Appendix 1). Personal factors referred to behaviours or internal states which influenced the experience of psychosis (n=18). Prior mental health problems and substance use was the most endorsed sub-theme (n=15) which contributed to feelings of being dismissed, resulting in managing their mental health alone. Some participants reported being unwell with mental health problems across the life course, and discussed how their previous mental health problems cumulatively impacted and explained their psychosis experiences.

‘I’ve been unwell the majority of my life, so as far as I can remember back … I was never really accepted for having mental health problems … it’s like what have you got to be depressed about?’ (Olivia)

Having a poor sense of self (n=7) was discussed through experiences of poor self-image, self-talk, and instances of self-blame. A strong sense of rejection in relationships and failure was reported by some participants (n=7), whilst others recognised stress as a contributing factor (n=4).

‘When I started working more directly with my experiences of hearing voices I realised that there was something around the inner dialogues I had with the voices and with my experience … My self-talk was rubbish’ (Victor)

Trauma factors (n=20) were predominately discussed within the context of childhood trauma and adversity (n=16) which was reported to contributed to the onset or experience of psychosis. Childhood trauma and adversity experiences included abuse, bullying, domestic violence, family issues, sexuality, parental mental health issues, bereavement, and crime, influenced an individual’s sense of self and relationships, which negatively impacted upon their experience of psychosis. Similar trauma experiences in adulthood (n=5) were reported but compounded through dealing with socioeconomic challenges. Others acknowledged psychosis as being a trauma.

‘I had a load of trauma, and I think that’s what I would call psychosis is trauma, rather than psychosis … there’s a reason why they see thing, hear things, to protect themselves’ (Robyn)

### 3.2 Objective 2: mechanisms of PTG in psychosis

Two mechanisms to PTG in psychosis were reported; Cognitive factors, and social/societal factors (Appendix 2). Interactions between factors were reported where perceived improvements in cognitive factors were associated with improvements in social/societal factors.

Cognitive factors were discussed by all 25 participants and were individual level changes, including having a greater understanding about one’s voices and coping strategies to deal with crises and to distract from voices. This was supported through family, support groups, and recovery colleges.

‘I started joining different groups, I decided to do some courses outside of the recovery college, all these things helped put more meaning into my life and helped me recover’ (Daniela)

Rumination (n=16) referred to thinking about past events and psychosis experiences that led to positive or negative impacts. Therapy supported deliberate rumination allowing reflection upon experiences. Yet, uncontrolled rumination or overthinking about experiences did not lead to gains. Rumination was a process that occurred after experiences of psychosis and for some lead to a changed outlook in life.

‘Before I had psychosis, I felt all this responsibility to live in a very specific way. Now that I’ve been able to sort through what thoughts are mine. it’s helped me a lot with knowing what I actually want’ (Palmer)

Acceptance of experiences (n=14) allowed for greater therapy engagement, medication use, and life exploration. Acceptance was reported by some to be necessary to be self-efficacious.

‘Accepting and reaching out to get help, I’ve realised that I have to do a lot of the work for reaching out to get help’ (Isla)

Making meaning of psychosis (n=9) was predominately discussed through its perceived purpose, including feeling less alone, psychosis as one’s life calling, or as a spiritual awakening. Friends and family supported meaning making through reframing techniques such as comparison (n=6) which encouraged a more positive tone. Constructive self-disclosure (n=3) allowed for reciprocity and sharing of ideas.

‘Having good days and remembering because when I do have a bad day, I’m like things have been better in the past, you know it can still get better. Reflecting on things that make me happy in the past, to try and keep me going and moving forward’ (Isla)

Social and societal factors were discussed by all 25 participants and referred to factors beyond the individual level factors. Themes discussed included healthcare and support services, personal relationships, resources, and cultural and spiritual factors. These factors compounded psychosis and intersectional experiences, and had helpful and unhelpful effects on PTG.

Healthcare and support services were discussed by all 25 participants. Care provided within hospitals (n=9) were seen to be a difficult experience, with only one participant reporting a positive contribution to growth. Services reduced capacity to meet specific needs (n=3) contributed to negative experiences such that feelings of being dismissed, being passed on to different teams, diagnostic changes, and clashes in explanatory models exacerbated the negative experience. Three other participants described their experiences in hospital as traumatic, leading to them feeling more vulnerable and hindered their recovery and growth. Views on medication (n=4) were mixed, with some reporting the unhelpfulness of medication whilst others indicated that finding the right medication or reducing the reliance on medication supported growth.

‘They were there to help me, but they didn’t help me. I felt more vulnerable, more at risk … I felt as if the team made it worse’ (Una)

Outpatient care was viewed more favourably in supporting growth compared with hospital care (n=18). However, a perceived lack of service resources, inequitable access and clinician disagreement left participants feeling vulnerable or at risk. A lack of access to resources (n=9) were predominantly finance and housing related. Whilst therapy was viewed to support growth (n=10), some recognised the privileged position of being able to pay for treatment which supported their growth (n=5). Having access to sufficient funds (e.g. benefits) was reported by some to alleviate day-to-day living strain.

‘I did get some good psychiatric help from a community mental health team. There was also a psychiatrist there, she was really good and she sort of help me when I went back to work.’ (Henry)

Personal relationships (n=21) supported and hindered growth. Relationships included partners, friends, family, children and the broader community. Thirteen participants positively described relational impacts. Personal relationships fostered a sense of belonging and connection, where participants felt supported and accepted considering their experiences. For a minority being in the presence of others was sufficient to combat feelings of loneliness and isolation, whilst some discussed how their relationships with others helped them to reframe the way they viewed their experiences. Negative consequences of personal relationships were discussed by two participants, where the feelings of not being accepted or believed and negative family dynamics contributed to the unhelpfulness of these relationships. Yet, two participants had a more nuanced view, such that different relationships led to different experiences, indicating that some relationships had positive impacts, yet others had more negative impacts.

‘My parents didn’t know as much, they never had to look after family, so therefore they didn’t know how to cope with me and sometimes they were not friendly. They wouldn’t listen to what I have to say, instead they would listen to their friends…’ (Emily)

‘My spouse has been a constant support … having a stable relationship made a massive difference … things that made it more difficult was probably my family, they didn’t understand at all…’ (Palmer)

Cultural and religious factors (n=6) on growth had a mixed effect. For some religious teachings supported making meaning of psychosis experiences, whilst for others religious institutions were unwelcoming and exacerbated stigma. The stigma associated with psychosis in some cultures was reported to lead to feelings of invalidation.

‘I think coming from the South Asian culture, mental illness isn’t discussed at all, and it’s frowned upon in society with a lot of stigma and it’s not considered as an illness or a disability. It’s just, as I say, part of your routine…’ (Una)

Activities meaningful to participants (n=15) were individual to each participant. General activities (e.g. dog walking, parenting, arts) supported growth through engagement in relationships and improving cognitive processes (e.g. disclosure, meaning making, self-efficacy). Giving back through volunteering in the community or using their lived experience of psychosis through peer support roles or professional training to become a health-related practitioner (e.g. psychologist, counsellor, open dialogue practitioner) also promoted growth. This gave individuals a sense of purpose and focus, which contrasted to periods when experiencing psychosis symptoms.

‘I think it was really valuable to use my experience in a positive way because it made me see something positive has really come out this … I found it quite empowering to be able to do that’ (Daniela)

### 3.3 Objective 3: outcomes associated with PTG in psychosis

The experience of PTG could be categorised into positive change or no change, which was based on a participant’s current state. Given the dynamic nature of psychosis, 24 participants were able to identify experiences of PTG, however at the time of interview, four participants discussed prior experiences of PTG and had difficulties recalling current PTG experiences. When prompted, most participants with difficulty identifying PTG experiences were able to discuss internal changes. One participant, however, reported only reported positive changes within their living circumstances. Positive change was characterised by the seven domains in the PROSPER model (Appendix 3).

Strengthening of personal identity was discussed by 18 participants. Changes in personal identity (n=10) through being able to differentiate between themselves and experiences of psychosis, where a participant’s identity consisted of more than psychosis experiences. Feeling stronger such that they felt more resilient, empowered, and finding strength in having dealt with the disruption of psychosis was a sign of growth (n=10).

‘I think all that suffering and the kind of terror that was in my mind, I think in a strange way, it does give you some freedom. ‘Cause you think, if I can survive that, I can survive anything.’ (Abigail)

Receiving support (n=9) was discussed through the ability to share experiences and receive support from others (e.g. friends, family, professionals). Facilitated through an improved ability to recognise the need for support, to be vulnerable, accepting support or receiving support following negative experiences.

‘It was hard, but then I also realized that … At the end of the day, I needed help because I had gone through a lot in solitude.’ (Quinn)

Recognising opportunities and possibilities (n=15) referred to participants embracing life, persevering and focusing on positive aspects. Engaging or re-engaging with education and employment (n=11) was considered meaningful, where some participants utilised their lived experience to support others.

‘I trained to become a peer mentor, because I received peer mentorship from different people, and then I just, they said that I should become one. So, I did a course on how to become a peer mentor.’ (Daniela)

Strategies for coping (n=12) referred to self-management techniques in managing symptoms and maintaining wellbeing. For some this involved working with symptoms and practicing self-compassion, whilst for others specific coping strategies mentioned included quitting substances, seeking joy, or having needs met.

‘Sometimes say like I’m on the bus and they [voices] are really on at me, I’ll kind of make a promise, and say I’m not gonna talk to you now, but I’ll talk to you later when I get home. And, and that kind of helps.’ (Abigail)

Experiencing a perspective shift (n=17) involved changes to one’s view of psychosis experiences. For some, there was a shift from viewing psychosis as unmanageable and feeling powerless, to feeling able to live well with psychosis, which consequently lead to hope for the future. Some participants experienced a change in behaviour (n=6) where a greater focus was placed on their own wellbeing and prioritisation for themselves and their needs. Participants made meaning (n=6) through finding a life goal or making sense of experiences.

‘A sense of purpose, strong sense of purpose has come from it, which I’m really grateful for … So, what happened was that I found a path where I actually felt, you know, that I was of use to other people.’ (Victor)

Enhanced emotional experience (n=11) was reported through having greater positivity, gratitude, joy and having greater empathy and compassion towards oneself.

‘I think the most important thing in life- well, not the most- one of the most important, is to have fun as well, and I think people underestimate that.’ (Abigail)

Improved relationships (n=18) was discussed through developing and improving new and existing relationships and through having access to greater support and understanding (n=9). For some participants this involved having stronger boundaries (n=9) or changes through parenting (n=3).

‘I think that part of the psychosis really helped me … because it’s through that, that I actually made the best friend.’ (Flora)

## 4 Discussion

The experience of PTG in psychosis is dynamic. Psychosis experiences were described as a disruption to their life. The psychosis experience was moderated by personal and trauma factors, with the experience of other mental health problems, a poor sense of self, and childhood/adult trauma reported as affecting their psychosis experience. Due to the dynamic nature of PTG experiences, some participants had difficulty identifying current experiences of PTG at the time of the interview. PTG experiences were reported across seven domains of positive change including personal identity and strength, receiving support, opportunities and possibilities, strategies for coping, perspective shift, emotional experience, and relationships. Mechanisms which influenced PTG experiences (positive or no change) included cognitive and social/societal factors. Cognitive factors were individually focused with participants reporting change in self-efficacy, rumination, acceptance, meaning making, and disclosure. Social and societal factors contributing to PTG included healthcare experiences, relationships, resources, and cultural and spiritual factors.

There are three major findings in this study. First, through a PTG perspective, the experience of psychosis can lead to either positive or no change. The indicators of positive change are individual in nature and consistent with the seven PROSPER domains. Given that the study inclusion criteria only included individuals who had perceived, self-reported experiences of PTG, the finding of no change at the time of interview was unexpected. Additionally, whilst participants discussed how psychosis experiences negatively impacted upon their life, discussion of negative growth/change was not discussed within interviews. This may be attributed to the inclusion criteria and the manner in which questions were phrased within the interview, limiting the types of experiences captured within the analysis. Yet, the sample included four individuals who reported difficulty identifying experiences of PTG at the time of the interview. Qualitatively, this finding indicates that experience of PTG can be stable or dynamic/non-linear, however the experience differs between and within individuals, such that the presence or absence at any given timepoint depends upon personal or life circumstances. A systematic review of quantitative longitudinal studies which examined trends in the development of PTG supports this finding (22). Of 22 studies included in the analysis, nine found stable PTG scores, six reported increases, and three reported a decrease over time, which differed depending upon trauma type, demographics, follow-up duration, and baseline PTG scores. Four studies examined PTG trajectories and found distinct patterns ranging from high-stable to low-decreasing. Whilst there are methodological differences between this study and the studies included in the systematic review, these findings indicate that PTG can be stable or dynamic in different individuals. It is implicated within the review, that this may be related to an individual’s coping style and use of active-adaptive (e.g problem focused) rather than maladaptive (e.g. emotion focused) coping strategies, as this is related to higher PTG. Some cognitive mechanisms identified in this study (e.g. rumination, acceptance of experiences and meaning made from experiences) support this finding. However, it should be acknowledged that psychosis has been widely documented as a challenging experience. Whilst our findings can provide hope that growth is possible, the negative consequences of psychosis (e.g. emotional toll, wider personal and social implications) can often be severe and long term. Taken together, we recommend a balance between addressing emotional and trauma needs with supporting growth.

Second, PTG has been predominately discussed as a single event trauma within the literature. The PTG in psychosis literature has referred to PTG occurring after the experience of psychosis. Yet, our findings on the experience of psychosis indicate a more nuanced experience. Participants discussed numerous factors which contributed to the trauma associated with psychosis (e.g. symptoms, hospitalisation, loss of life trajectory) which could be exacerbated through intersectional experiences. This indicates that the trauma of psychosis is cumulative involving a multi-faceted experience. Clinically, this suggests that there is a need to consistently revisit conversations surrounding PTG with awareness of its dynamic nature. The focus of support should be tailored towards the needs and goals of individuals. As iatrogenic harm from healthcare was reported by participants, awareness of the potential impact clinicians can have requires reflective practice.

Third, mechanisms of action in understanding PTG have been described as an individual process and research into PTG in psychosis have predominately focused on individual level mediators (e.g. meaning in life, effective coping skills (16, 17), and clarification of core beliefs (18)). However, the findings indicate that several relational and societal factors are implicated in supporting or hindering growth processes. Whilst there are significant mental health studies that have examined the social determinants of health (23), the role of social and societal factors in mediating the relationship between psychosis and PTG has not been explored. Quantifying the impact of intersectionality through multiple disadvantage could also help to ascertain priorities for mental health services. Additionally, based on participant responses, the experience of PTG appeared to be mediated by an interaction in changes between individual level and social/societal level factors. Inclusion of both individual, social and societal factors within mediation/moderation models may provide a more nuanced understanding of factors which can best support PTG.

There are two clinical implications associated with the findings. First, clinicians working with people with psychosis should monitor and support experiences of PTG. Whilst this is a small qualitative study, findings indicates that PTG may be a common experience. Previous cross-sectional survey studies found that people with psychosis who use mental health services experience report moderate levels of PTG (15). However, it is important to consider that not all individuals will experience PTG. One participant in this study reported no internal PTG related changes, despite reporting changes in external life circumstances. Second, efforts to promote PTG in people with psychosis requires intervention at different levels. Individual level interventions can target mechanisms involving cognitive factors. For individual interventions, an initial focus on promoting active-adaptative coping strategies (e.g. problem focused coping strategies) might be most useful in promoting PTG. Studies have indicated that active coping processes, such as rumination and positive reframing (both indicated as mechanisms within this paper) support PTG (24). Yet, social/societal factors, may be better addressed through population-level public health interventions or prevention programs.

### 4.1 Strengths and limitations

There are four strengths and limitations associated with this study. First, this is the first study examining experiences of PTG in psychosis which recruited a heterogenous sample. Participants were recruited from beyond secondary/inpatient mental health services and 48% of individuals were not of white British ethnicity. Moreover, the sample included 24% of individuals who had self-reported psychosis experiences rather than a clinical diagnosis of a psychosis spectrum disorder. Whilst this may reduce the clinical utility of the study which would have allowed for greater generalisability precision and diagnostic specificity, from an equality perspective, this allowed for the inclusion of a more diverse group of individuals and experiences. Making meaning from experiences was identified as a mechanism of action in this study. It is plausible that culture influences can shape the way that a person makes sense of their experiences. The inclusion of people with a range of identities and different demographic factors shapes findings to be more inclusive, representative, and generalisable to the general population. The findings also extend current PTG in psychosis knowledge to understanding the experiences of individuals who do not use mental health services. The existing PTG in psychosis literature solely focuses on the experiences of individuals who are recruited participants through clinical services (25). However, it is known that people can recover from mental health problems outside of services (26). Additionally, whilst mental health services have been identified as one source of support in promoting PTG, it is likely that people continue to experience PTG when they no longer require services. Whilst it is acknowledged that our sample was overrepresented by individuals who are highly educated and female, these findings provide a more ecologically valid representation of the process of PTG in psychosis.

Second, the PTG literature recommends conducting longitudinal research, yet we adopted a cross-sectional design involving participants recalling PTG experiences. Whilst we acknowledge that individuals are experts of their own experience, there may have been some element of recall bias amongst the responses. There are two potential areas for future research. Future PTG in psychosis research could consider adopting longitudinal designs which involve individuals who are at high risk of psychosis but may not yet show symptoms to understand the trajectory. Additionally, examining whether endorsement of the PROSPER framework domains changes over time can help to illuminate the stable vs dynamic/non-linear experience of PTG over time. This may involve a combination of longitudinal qualitative follow-up studies and quantitative work to understand whether there are patterns of PROSPER domain endorsement across recovery stages. Whilst this may provide clinicians an indication of possible areas for discussion, collaboration with individuals to understand their goals is crucial. Third, the involvement of the LEAP throughout the study ensures that the design of the study and interpretation of findings is grounded in lived experience. While the nature of qualitative analysis is inherently subjective, discussion and triangulation with the LEAP aimed to mitigate any misinterpretation of data.

Fourth, our recruitment and interview were primarily online, introducing a selection bias against those without technological access, which must be considered against the context that a higher proportion of people with mental health conditions experience digital poverty (27).

## Data Availability

The datasets generated and analysed during the current study are not publicly available as participants did not provide consent for open data sharing, but can be made available from the corresponding author on reasonable request. Requests to access the datasets should be directed to fiona.ng@nottingham.ac.uk.
