# Correction: Development of a process model of posttraumatic growth in psychosis: a qualitative study

**DOI:** 10.3389/fpsyt.2026.1849347

**Published:** 2026-04-13

**Authors:** Frontiers Production Office

**Affiliations:** Frontiers Media SA, Lausanne, Switzerland

**Keywords:** mechanisms, mental health, outcomes, positive change, posttraumatic growth, psychosis, qualitative, recovery

In the original version of the article, the affiliation list was erroneously given as:

“1. Department of Community Mental Health, University of Haifa, Haifa, Israel

2. Evaluating Positive Changes in Psychosis (EPOCH) Lived Experience Advisory Panel, Nottingham, United Kingdom

3. Ono Academic College, Kiryat Ono, Israel

4. Department of Psychiatric Rehabilitation and Counseling Professions, School of Health Professions, Rutgers University, Newark, NJ, United States

5. Nord University, Faculty of Nursing and Health Sciences, Health and Community Participation Division, Namsos, Norway”

This should be:

“1. School of Health Sciences, Institute of Mental Health, University of Nottingham, Nottingham, United Kingdom

2. Evaluating Positive Changes in Psychosis (EPOCH) Lived Experience Advisory Panel, Nottingham, United Kingdom

3. Ono Academic College, Kiryat Ono, Israel

4. Department of Community Mental Health, University of Haifa, Haifa, Israel

5. Department of Psychiatric Rehabilitation and Counseling Professions, School of Health Professions, Rutgers University, Newark, NJ, United States

6. Nord University, Faculty of Nursing and Health Sciences, Health and Community Participation Division, Namsos, Norway”

In the original version of the article, author “Yael Mazor” was erroneously assigned to affiliations 1, 3. This should be “3, 4.”

In the original version of the article, author “David Roe” was erroneously assigned to affiliations 1, 4. This should be “4, 5.”

In the original version of the article, author “Mike Slade” was erroneously assigned to affiliations 1, 5. This should be “1, 6”.

The final author list therefore reads:

“Fiona Ng^1^*, Benjamin-Rose Ingall^1^, Susie Booth2, Matt Geeleher^2^, Julian Harrison^2^, Mark Holden^2^, Felix Lewandowski^1^, Yael Mazor^3,4^, David Roe^4,5^ and Mike Slade^1,6^

^1^School of Health Sciences, Institute of Mental Health, University of Nottingham, Nottingham, United Kingdom, ^2^Evaluating Positive Changes in Psychosis (EPOCH) Lived Experience Advisory Panel, Nottingham, United Kingdom, ^3^Ono Academic College, Kiryat Ono, Israel, ^4^Department of Community Mental Health, University of Haifa, Haifa, Israel, ^5^Department of Psychiatric Rehabilitation and Counseling Professions, School of Health Professions, Rutgers University, Newark, NJ, United States, ^6^Nord University, Faculty of Nursing and Health Sciences, Health and Community Participation Division, Namsos, Norway”

The original version of this article has been updated.

