# Supplementary material for: Development of a process model of posttraumatic growth in psychosis: a qualitative study
Source: Front Psychiatry. 2026 Mar 30;17:1774487. doi: 10.3389/fpsyt.2026.1774487 (PMC13112059; doi:10.3389/fpsyt.2026.1774487)
Supplement: Supplementary file 1 [file Table1.docx]

Appendix 1: Moderators of PTG in Psychosis (n=25)

|  | **Definition** | **Example** | **Frequency** |
| --- | --- | --- | --- |
| **Moderator 1: Personal Factors** | | | **18** |
| Mental Health Problems and Substance Use | Discussion surrounding mental health problems prior to experiences/diagnosis of psychosis and effects of substance use. Includes discussion about learning difficulties and neurodiversity. | “It's hard to say whether it was too much alcohol, but I was, was drinking quite a lot as well, which it didn't seem unusual, um, being with, with the friends that I was, I was around. Everyone was kind of drinking and going on nights out. I really don't think any of those things helped me.” – Kelly  “I even had anxiety and depression because of my diagnosis and like, um, I don't know if it was linked or if it was two separate things, but I was also very like poorly.” - Lilia  “I was dyslexic when I was younger, couldn't read or write til I was 14 and therefore pushed like hell.” - Marcus | 15 |
| Sense of Self | Aspects of self which impacted psychosis experiences. Includes; self-esteem, self-image, self-worth, personality traits, values, and identity | “So I kind of understand like, um… Having very critical voices and stuff, I think is, um… Partly about my self-esteem, but also, I think also, you know, from sort of times of being bullied or…” – Zara  “So my personality is very competitive, A, outgoing. And I thought everyone valued by what you do and what you achieve.” - Marcus | 7 |
| Social Difficulties | Social difficulties experienced prior to psychosis, including difficulties relating to others, maintaining relationships, setting social boundaries, or social isolation. | “Because before that [the breakdown], I'd been in relationships and things where, well now looking back, they're incredibly controlling and manipulative really.” – Olivia | 7 |
| Stress | General life stressors contributing to experiences of psychosis | “…Leading up to the first episode, I was doing my PhD, and I was working at the same time, so I was working quite a lot. I was working in the NHS. Um… I was also kind of moving house. Um… And that was kind of stressful, because I was leaving a flat that I'd lived in with my ex-partner, and it had been a quite difficult break up, like a long time before, but I hadn't moved away from the flat…” - Nia | 4 |
| **Moderator 2: Trauma Factors** | | | **20** |
| Childhood Trauma and Adversity | Discussion of adversity and trauma experienced in childhood prior to the psychosis. | “As a child growing up, um… And I experienced many traumatic events, such as, um… I was a victim of a… Crime but didn't have PTSD. I had a… Very, very traumatic experiences where I felt very, um… On edge.” – Una | 16 |
| Adulthood Trauma | Discussion of trauma experienced in adulthood prior to the psychosis. | “So basically I was… Had a load of trauma, and I think that's what I would call psychosis is trauma, rather than psychosis. And because everybody has got, there's a reason why they see things, hear things, whatever. Uh, to protect themselves.” – Robyn | 5 |
