# Supplementary material for: Development of a process model of posttraumatic growth in psychosis: a qualitative study
Source: Front Psychiatry. 2026 Mar 30;17:1774487. doi: 10.3389/fpsyt.2026.1774487 (PMC13112059; doi:10.3389/fpsyt.2026.1774487)
Supplement: Supplementary file 2 [file Table2.docx]

Appendix 2: Mechanisms of PTG in Psychosis

| **Sub-theme** | **Definition** | **Example** | **Frequency** |
| --- | --- | --- | --- |
| **Mechanism 1: Cognitive Factors** | | | |
| Self-Efficacy | A person’s belief in their ability to cope | “I decided, right, now, from now on, I'm going to be on the recovery journey and everything I do is gonna be for my recovery, I'm not gonna do anything that is not about my recovery, but I was that strict with myself, because I literally had nothing left, because I was so unwell.” – Daniela | 18 |
| Rumination | Deliberate or unintentional processing of experiences which involve considering the impact and emotional responses of experiences. Deliberate rumination may be supported through healthcare and support services (e.g. psychotherapy) | “So my main problem was at night, especially when I was just alone… I had sleepless nights actually, because I'll just wake up at night and then I'll just be thinking, why did they choose me over the rest? And, why they, OK, why my mother not the other mothers or anything?” – Flora | 16 |
| Acceptance | Acceptance of self, the experience of psychosis and/or impact of psychosis | “So what it's helped me to do, is understand that my sense of self is like, that of somebody who has got a mental health issue, and like quite severe one of that. But is able to manage it quite effectively if I'm accepting of it, it's when I'm not accepting of it that it kind of just goes boom, like it's a huge elephant in the room.” – Olivia | 14 |
| Meaning Making | Developing a personally meaningful understanding or making sense of experiences. | “I guess a belief that it, that the experience is something greater than yourself and something that, you know, like a spiritual experience.” – Nia | 9 |
| Learning and Information Seeking | Learning about mental health, psychosis, and recovery from medical or alternative perspectives as influencing their experience of positive change. | “The main thing I think that really helped me was, you know, like I understood more about, like, you know, what I was going through and, you know, what the disorder is […] I did like a lot of background, like, you know, research about it to find out more about the thing.” – Lilia | 7 |
| Comparison and Reframing | Comparing one’s life or situation to another person’s or to another time in their life. Changed approach to viewing an experience or situation | “I think sort of having like… Some good moments as well, have helped me realize that, like oh, you know, you actually you can feel happiness, you can get on with your friends, you can still feel love. You can still feel like safe. You can go out and be happy, you can go out and not feel like scared or vulnerable. You can get on the bus, and you don't have, like nasty thoughts.” - Isla | 6 |
| Disclosure | Revealing experiences of psychosis to others | “Yeah, just being quite honest, I think, with my mental health I think is quite helpful. Although a lot of people still feel, oh, you shouldn't talk about that, or that's shameful, but for me, I'm quite happy to talk about those things and it's helpful for others, innit, that's, that, I guess that's what it is. Yeah. And doing things like this, you know, that's hopefully helpful for other people, so. Um, I guess they're the positives from, from that, yeah.” - Henry | 3 |
| **Mechanism 2: Social and Societal Factors** | | | |
| Healthcare and Support Services | Referring to the role of formal healthcare & support services, such as outpatient care, hospitalisation, medication, and social services on PTG. | *Inpatient and Outpatient care*  “I think it was through therapy. I did some different therapies, talking therapies, and I think that early on this psychologist realised what was going on, but it took me a bit of a while to understand what the meaning was.” – Daniela  *Hospitalisation*  “One other thing that made it more difficult, I don’t want to focus too much on the negative, was in the mid 80’s, when I was in a psychiatric hospital, there was a nurse there who told me there was no hope for me.” – Chris  “I think when you’re psychotic, your mind tries to make you have these ideas that you do matter and I think ironically the problem is you’re going to hospital with these feelings and the message you get is you don’t matter. So it’s kind of really unhelpful to have your mind try make you feel important and make you feel that you are something, but unfortunately what you’re getting in hospital is, oh you’re just a nutter, you’re mad, here’s some medication.” – Abigail  *Medication*  “All the mood stabilizing stuff that I'm on, has supported and enabled me to say yes to all sorts of things, whereas I think before… Well, I wouldn't have known I could. So you know, I can't say too many bad things about psychiatry…” – Robyn  “I’ve been cajoled into taking antipsychotics, I’ve been forcibly injected the first time. Um… with a mixture of antipsychotic and another sedative, I think, I can’t remember, it was a long time ago. Um… but yeah, I mean, it seems antidepressants, basically what is known about the actual drug effects is that they’re, the antidepressants are sedatives as well.” - George  *Social Services*  “I did get help from the social service, as to, regards her being with her dad, and her growing up. So, I am not ungrateful” – Emily  “I had it was very, very difficult with social services when [child’s name] was little, but we kept her and you know, it's, it's all okay in the end, but it was very hard. I found it very discriminatory, the whole thing.” – Abigail | 25 |
| Personal Relationships | The impact of connecting with others in supporting PTG, including the discussion about the types of personal relationships (family, support network, partner, friends, child) and the impact these personal relationships, with acknowledgement that different relationships have different dynamics | “I like that community. I like that feeling of belonging, because I think when you become really psychotic and very unwell, and I will use the term unwell because that's how it feels sometimes, to be unwell.” – Abigail  “My spouse has just been like a constant support. Having a stable relationship made a massive difference, even if they weren't like able to do things that like actively give me coping techniques, just having stability was a massive, massive help. Um, things that made it more difficult was probably my family, they didn't understand at all.” - Palmer | 21 |
| Meaningful Activities | Personally meaningful activities or engagements which supported PTG. These could involve giving back to a community, the sharing of mental health lived experience or general activities | “I did some other courses at the local comprehensive technical college. I did a GCSE in Spanish, and I trained to become a peer mentor, because I received peer mentorship from different people, and then I just, they said that I should become one. So, I did a course on how to become a peer mentor, and that was really helpful because I could see that other side of the coin, like I could see why people had been saying certain things to me and how that had helped me, because they were training me to be the listener and support that other person.” – Daniela  “Walking with my dog in nature. Because I was not working, I was able to get my dog, spent a lot of time with my dog, while I was like, mulling all these ideas over.” – Kelly  “Interaction with the society and with the outside environment that's sort of improved cause I stopped going outside. I stopped like you know, I just shop online, but like in the past year or so started to go out more, you know, participate in like hobbies.” - Lilia | 15 |
| Resources | Availability and access to resources including financial, housing and immigration related | “But obviously what made it difficult, I have to look for money. I have the housing struggles.” – Tyra  “I think the positive changes are, you know, the mental health trusted team, I think given my own flat where I am living independently, but with support. That's a positive change. If services that didn't, had not, had not geared in and hadn't supported me at the time, um, I don't know like, how could, I was supported financially with my benefits, I was given benefits and I could claim them, as a result, I was giving support in that way and form.” - Una | 9 |
| Culture and Spirituality | The influence of an individual’s culture, religious upbringings, religious teaching and stories, and personal spirituality on PTG | “Religious teaching. Has… Confirmed it like in that… Sort of…  Belief, as well, like in my religion, where we know that are other creations that exist that naked human eye in this world, anyway. So, like knowing that from a religious perspective or angle, has, yeah, influenced that.” – Joe | 6 |
