# Supplementary material for: Development of a process model of posttraumatic growth in psychosis: a qualitative study
Source: Front Psychiatry. 2026 Mar 30;17:1774487. doi: 10.3389/fpsyt.2026.1774487 (PMC13112059; doi:10.3389/fpsyt.2026.1774487)
Supplement: Supplementary file 3 [file Table3.docx]

Appendix 3: Indicators of PTG in Psychosis

| **Sub-theme** | **Definition** | **Example** | **Frequency** |
| --- | --- | --- | --- |
| **Indicator 1: Personal Identity and Strength** | | | **18** |
| **Indicator 1a: Personal Identity** | | | **10** |
| Differentiating self and experiences | Ability to separate one’s identity from experiences of psychosis | “I think sort of the older I've gotten, I've kind of realized that, like, my mental health doesn't have to be my personality, if that makes sense.” – Isla | 3 |
| Self-knowledge and understanding | Development of a deeper or more authentic understanding of themselves, such as increased awareness of their needs, limits, boundaries, and beliefs. | “I understand my life a lot better now, through reflection and therapy and the voices, and I understand where my boundaries are. So, I know what I should and shouldn't do, what I should and shouldn't say, who I should go to. I'm much more in control of my life now.” – Daniela | 8 |
| **Indicator 1b: Personal Strength** | | | **10** |
| Empowerment | Experiences of becoming empowered, and of self-empowerment. These both relate to the experience of becoming stronger, more confident, and feeling that one has power to make changes in life. “Empowerment” is the experience of having been supported towards these outcomes, such as by a therapist, by family, or by friends. “Self-empowerment” refers to the experience of being the active agent in this process. | “I think the change has been where I've learned for myself to, you know, to empower myself, you know, to… To… The ability where I can be in a position to, to question, to refuse, to take my own initiative, what I feel is appropriate for, um… That’s empowerment its given me, like to take the steps in order to improve my own recovery.” – Una | 4 |
| Self-Efficacy | Experiences of feeling more competent and in control of one’s life. Includes feeling able to take control of their situation and make changes, though identifying their current situation and their desired future situation, and setting realistic goals and working towards them. It also includes feelings of determination in finding a solution, feelings of responsibility for ones own path, and feelings of being disciplined in their seeking of goal achievement. | “I got that strength that I have to make it on my own. Since I have no one to motivate me, I also, again, and have no connection or anything. So, I just have to stand on my own. Make my own life, in future start my own family, so that at least that family name will never die, just like that.” – Flora | 6 |
| Resilience | Strength from having coped with a disruptive experience before, thus providing the security that they can cope again in future. | “It makes me feel more resilient, having like, know that I've been through this before and I can get through it […] But actually, I know I've been there, I can do it again, and although I absolutely don't want it to happen again, it was still, yeah like, it makes me feel like I've been through something and it's empowering to know I've been through it.” – Bella | 7 |
| **Indicator 2: Receiving Support** | | | **9** |
| **Indicator 2a: Openness to Support** | | | **9** |
| Openness to Sharing | Experience of becoming more able to speak openly with others | “It was actually very difficult to actually reach out to people to get help. Like, the sort of like trying not to… Um, sort of that expose myself for trying to be that, that, you know, that figuring out how to be that vulnerable person, who has to share these kind of difficulties of having with other people, was one of like the hardest things ever, I feel like.” – Lilia | 7 |
| Openness to Receiving Support | Experience of feeling more able to ask for or receive support from others | “OK, it was hard, but then I, I also realized that… At the end of the day, I needed help because I had gone through a lot in solitude.” – Quinn | 6 |
| **Indicator 3: Opportunities and Possibilities** | | | **15** |
| **Indicator 3a: Embracing Life** | | | **7** |
| Perseverance | Sense of determination to engage with life despite the challenges of their experiences. | “I think sort of like, the part that's, like, kept me going is, like, people saying that like, despite what you're going through, like, things can get better and I guess that's sort of what I'm looking forward to, like… just, like, giving life another go and just trying to make the most out of it. And I think sort of like, not letting things, like, ruin my life.” – Isla | 5 |
| Positivity | Adoption of a general sense of positivity and optimism towards life. Life is experienced as good and as precious. | “I saw life as, life was better. And, and, and actually, yeah, like time, time is precious and should not be wasted.” – Wilma | 2 |
| **Indicator 3b: Meaningful Activity** | | | **11** |
| Employment and Education | Refers to the (re)engagement with employment or education where this is experienced as meaningful for the individual. | “I've just completed this degree that I've been working on for like 15 years, so… Yeah. Yes, I'm, I'm hoping to do an occupational therapy degree […] so I’d want to sort of hopefully get that course in place this year. So that's the hope.” – Bella | 3 |
| Mental Health Work | Refers to activities engaged in or opportunities which have arisen which allow the individual to support others through utilising their lived experience. This includes paid employment, volunteering, peer support, and activism. | “From… Developing awareness, and developing relational skills, and from learning to articulate my experiences and articulating understandings, and then articulate what has help, has sort of led me into a role in within teaching and facilitating training and things like that, which I would never, you know, I always wanted to be a dancer, performing artist, or, so I've ended up doing something completely different in my life, but it feels very meaningful.” – Victor | 10 |
| **Indicator 4: Strategies for Coping** | | | **12** |
| **Indicator 4a: Symptom Management** | | | **4** |
| Self-Compassion | Regarding the self with more compassion and kindness as a strategy for coping. | “Yeah, that shift in perspective, and that shift, you know, being reminded or being encouraged or supported to be more compassionate, be more accepting, maybe take things less seriously or take some things more seriously that you need, you know.” – Victor | 3 |
| Working with Symptoms | Working with symptoms, as opposed to symptom repression. | “So, sometimes say like I'm on the bus and they [voices] are really on at me, I'll kind of make a promise, and say I'm not gonna talk to you now, but I'll talk to you later when I get home. And, and that kind of helps, it doesn't always work, because strategies in any- whether it be business or whatever, strategies don’t always work 100%. But it's nice to have that strategy and it does, it does help.” – Abigail | 3 |
| **Indicator 4b: Wellbeing Maintenance** | | | **11** |
| Emotional Openness | Refers to expressing ones emotions honestly, as opposed to repression. | “So, I tell myself, let the, let the tears flow. It will dry up one time and I'll be fine. But it takes a very, very long time. So, from then on, whenever I realized that the tears start to flow, I just get on with my life.” – Samir | 3 |
| Energy Management | Refers to recognising and respecting the boundaries of ones energy. | “I create a bank balance. So it's an artistic activity, fold a piece of paper in two and then, what are the things that nourish you, and what are the things that deplete you. One on each side and drawings of those. And then looking at that as a bank balance and, you know if you've got a, a financial bank balance, you work hard not to go overdrawn and to make sure that you're in credit, and you hope that you'll be, you know, your credit will build up and build up and build up.” – Yvette | 2 |
| Meeting Needs | Refers to engaging with ones basic needs. | “I've tried to make a real change, like even stuff like making the bed in the morning, like instead of sitting in that bed all day, I kind of make the bed and then, you know, just throwing away rubbish, clearing up stuff off the floor so I can actually see my floor and walk through my room, I think. It's like, silly as it sounds, just being able to see the floor and walk without falling over or hitting something. Yeah, so it's, it's a nice feeling.” – Isla | 2 |
| Quitting Substances | Refers to abstaining from substance use. | “I avoid like anything like alcohol or drugs that kind of like would affect how I view things. I'm still quite, uh, cautious about anything that might alter my thinking.” – Palmer | 3 |
| Seeking Joy | Refers to prioritising the importance of seeking joy and having fun. | “I think the most important thing in life- well, not the most- one of the most important, is to have fun as well, and I think people underestimate that.” – Abigail | 3 |
| **Indicator 5: Perspective Shift** | | | **17** |
| **Indicator 5a: Changing Views** | | | **13** |
| Regarding Experience | Change in how they view their psychosis experience, such as changing in their understanding of it as something unmanageable to manageable, from "no hope" to hope for recovery. This includes the realisation of having psychosis, a move from "this is normal" to "not everyone experiences these things". | “I think it's part of like seeing it holistically, I, I can understand from like many, many lenses, what, what it could be. But I think, I think the spiritual, um, explanation has been quite important in my recovery, over the, the medical one.” – Kelly |  |
| Regarding Life | A change in the way they view life as a whole, such as seeing life as more valuable, worth living, or the sense of having a second chance. | “I saw life as, life was better. And, and, and actually, yeah, like time, time is precious and should not be wasted.” – Wilma |  |
| **Indicator 5b: Changing Behaviour** | | | **6** |
| Focus on Wellbeing | Change in behaviour to focus on ones own wellbeing or recovery journey. | “Because of what happened, the way I had nothing left, and you know, I said like, I tried to put things in which was supportive of my health. So, now most of the time, I'm doing stuff which is good for me, because that's what I decided I had to do, because when I was really ill, the voices were like 100% loud and 100% of the time, and I couldn't live like that.” – Daniela | 4 |
| Self-Prioritisation | Change in behaviour to prioritise the self over others where one previously did not. | “So, I've wrestled with that as well, with the, with that kind of selfishness, selflessness, dichotomy and the shame, the shame has been a big, big one around that. But also it was like, I knew that, for example, I was so, I was so stuck in putting my children first to the point where I had nothing left, that then I cracked up and didn't live with them for 14 months. So, when I got them back, I had to put myself before. Then it was like I had to be OK. It's that. Put your own mask on first.” – Yvette | 2 |
| **Indicator 5c: Meaning Making** | | | **6** |
| Meaning in Experience | The experience of having found meaning or value in their experiences of psychosis or its consequences | “It's just up to a person to accept the, the changes that have, that have happened in their lives, and to make something, just look at the positive side of it, I looked at the Christianity part of it, and I just know that it was God's purpose.” – Flora | 5 |
| Meaning in Life | The experience of having found renewed meaning or purpose within their life. | “I thought, wow, could I in any way, with the rest of my life, support prisoners or become involved in criminal justice, or, um, support the work of Sister Helen and maybe, maybe I went through psychosis to help people that are struggling in some way, and, um, with their mental health and criminal justice system.” – Kelly | 4 |
| **Indicator 6: Emotional Experience** | | | **11** |
| **Indicator 6a: Enhancing Emotional Experience** | | | **7** |
| Focus on Positive | The experience of focusing on the positive aspects of ones life or the positive outcomes of ones experience. | “I have that luxury, I suppose, where I, I kind of, it sounds- I mean, people shout me down for saying this, that going to hospital was a luxury, but that I was in that, kind of milieu where I could talk about feelings, it wasn't shameful to feel depressed, it wasn't shameful to say I can't work. These things were, were, were, were kind of- and, and I was given a lot of time and attention, […] So, I sorted all that shit out a long time ago, but she's still going through it. She's 52, and she's kind of has never resolved any of this, so in that sense I was, I was very lucky, you know.” – Abigail | 7 |
| Gratitude | The experience of reflecting on good aspects of life, being thankful for what one does have, for example, being thankful of support received. | “Trying to like look at, like, being more grateful, like having gratitude, you know.” – Lilia | 3 |
| **Indicator 6b: Empathy and Compassion** | | | **6** |
| For Others | These individuals described having more compassion, empathy, and understanding for others. This is shown through behaviours such as being more accepting of others, making efforts to understanding their points of view or things they have been through. | “I think it's helped me accept other people as well, you know, accept all different walks of life, you know.” – Henry | 5 |
| For Self | These individuals described developing more compassion towards themselves, through extending themselves kindness through difficulty. | “There’s this classic thing of, uh… Trying to talk to yourself like a friend would, because we never, we never seem able to talk to ourselves the way we would talk to our friend, or our friend would talk to us. So just that… Yeah, that shift in perspective, and that shift, you know, being reminded or being encouraged or supported to be more compassionate, be more accepting.” – Victor | 2 |
| **Indicator 7: Relationships** | | | **18** |
| **Indicator 7a: Developing and Improving Relationships** | | | **9** |
| New Relationships | These individuals described the formation and development of new relationships as a direct or indirect result of their experience with psychosis. | “I was very lucky, I mean, not long after my very first admission, I met my husband, [name], and we've been together 30 years, so and, and also without, if, if I hadn't had that experience, I wouldn't have [name] and I wouldn't have my daughter.” – Abigail | 2 |
| Strengthening Relationships | These individuals described experiencing their pre-existing relationships as stronger or as deeper after their experiences with psychosis. | “I think that part of the psychosis really helped me, because if it was not for it, I will not have identified my strengths, because it's through that, that I actually, I actually made the best friend.” – Flora | 7 |
| **Indicator 7b: Relationship Boundaries** | | | **9** |
| Improved Social Understanding | These individuals described having an improved social understanding as an area of growth, such as more clearly understanding social relationships, understanding the motivations of others, and developing social skills. | “My experience of psychosis made me realise that more is going on beneath the surface and to question people's motives. Which has been a positive for my life.” – Daniela | 3 |
| Establishing Boundaries | These individuals described their experience of setting limitations, expectations, and boundaries onto their current and/or future relationships in order to prioritise their wellbeing. | “I guess… Sharing things that I'm not necessarily wanting to share or, that, that, that's something that's, um, obviously… You know, only share things that I'm comfortable sharing with. That's kind of a, a lesson learned.” – Henry | 5 |
| Ending Relationships | These individuals described the deliberate decision to distance themselves from, or end, certain relationships for their own wellbeing. | “When it was really bad, I did not understand, I just knew that I was having problems with people. But as soon as I cut them all out of my life, my life got better, because I realized I don't have to see them anymore, I don't have to involve them in my life.” – Daniela | 3 |
| **Indicator 7c: Parenting skills** | | | **3** |
| Improved Parenting Skills | These individuals described the ways in which their experience of psychosis and/or the experiences of managing their mental health has improved their parenting skills. | “So I've got a 20 year old […] and people sort of go, ohh, despite everything, she's still turned out really well, and I go hang on a minute, hang on a minute, maybe because of our mental health issues, this has been a healthy environment for her to grow up in.” – Abigail | 3 |
